# Recommendations on hip fractures

**DOI:** 10.1007/s00068-016-0684-3

**Published:** 2016-07-14

**Authors:** K. Wendt, D. Heim, C. Josten, R. Kdolsky, H.-J. Oestern, H. Palm, J. B. Sintenie, R. Komadina, C. Copuroglu

**Affiliations:** 1 Trauma Surgery, University Medical Center Groningen, P.O. Box 30001, 9700 RB Groningen, The Netherlands; 2 Hohmad Privatklinik Thun, Hohmaddstrasse 1, 3600 Thun, Switzerland; 3 Klinik für Orthopädie, Unfallchirurgie und Plastische Chirurgie, Universitätskliniken Leipzig, Leibigstrasse 20, 04103 Leipzig, Germany; 4 Departmernt for Emergency Surgery, Medical University of Vienna, AKH Wien, Währinger Gürtel 18-20, 1090 Vienna, Austria; 5 Schubertstrasse 12, 29223 Celle, Germany; 6 Department of Orthopaedics, University Hospital Hovidovre, Kettegard Alle 30, 2650 Hovidovre, Denmark; 7 Department of Surgery, Elkerliek Ziekenhuis locatie Helmond, Wesselmanlaan 25, 5797 HA Helmond, The Netherlands; 8 Department of Surgery, Teaching and General Hospital Celje, Oblakova Ulica 5, 3000 Celje, Slovenia; 9 Department of Orthopaedics and Traumatology, Faculty of Medicine, Trakya University, Balkan Yerleskesi, 22030 Edirne, Turkey

## Introduction

Hip fractures among the elderly are one of the major fragility fractures in terms of quality of life, health outcomes and medical costs [1]. Since mortality and morbidity are high, hip fractures have a direct impact on public health [2] and are one of the main reasons for disability [3].

Increases in age-adjusted incidence of falls with accompanying deterioration in age-adjusted bone quality may explain the reason for osteoporotic hip fractures among the elderly [2].

According to United Nations records from 2009, the average lifetime of human beings was 56 years in 1970; by 2000 it rose to 65 and by the year 2050 it is expected to be 75.5 years (73.3 for men and 77.9 for women) [4]. According to some epidemiological studies, there were 1.66 million hip fractures worldwide in 1990. Epidemiological projections estimate these annual figures to rise to 6.25 million by 2050 [2]. In another epidemiological study, the total number of hip fractures in 1990 was found to be 1.26 million; this is estimated to approximately double to 2.6 million by 2025 and to 4.5 million by 2050 [5]. As advances in medicine and healthcare awareness increase, life expectancy at birth and lifetime spans will rise exponentially.

Management of hip fractures requires a wide spectrum of approaches, from prevention to postoperative care [6]. The socioeconomic impact of hip fractures is increasing on a worldwide scale, and there is a need to develop preventive strategies [5] as well as evidence-based treatment protocols to minimise the enormous social burden of these fractures.

Given that hip fractures are common, costly injuries with a complex treatment journey that is complicated by comorbidities in the elderly patient group, building clinical recommendations is an important and challenging topic if one considers that infrastructures do vary among European countries. These recommendations are therefore proposals for medical treatment in typical situations, and do not constitute legally committing rules to be observed.

### ESTES study group, proximal femur

**Table:** 

Cem Copuroglu	Turkey
Dominik Heim	Switzerland
Christoph Josten	Germany
Richard Kdolsky	Austria
Radko Komadina	Slovenia
Hans-Jörg Oestern	Germany
Henrik Palm	Denmark
Jan Bernard Sintenie	The Netherlands
Klaus Wendt	The Netherlands

The European Society for Trauma and Emergency Surgery (ESTES) Study Group was formed in 2014 with the aim of developing ESTES recommendations on proximal hip fractures. After a review of the recent literature and already existing guidelines in several European countries the members of the study group wrote a concept of the different parts of the recommendations. On a consensus meeting in September 2014 in Frankfurt a definitive version of the recommendations was formulated and agreed by all study group members. The recommendations on hip fractures are approved by the ESTES board.

### Patient group and aims

These recommendations focus on elderly people with a minor trauma of the proximal femur:

1. Extra-articular pertrochanteric/subtrochanteric fractures (AO classification 31 A1–3).
2. Femoral neck fractures (AO classification 31 B1–3).

Minor trauma can be a fall indoors or outdoors from a standing height. Restoring the level of activity is the main treatment goal. To reach this goal a multidisciplinary approach is necessary. A trauma (orthopaedic) surgeon, anaesthetist, geriatrician and emergency physician can be part of the team. The coordinator should be a trauma (orthopaedic) surgeon [7]. He has the ability to overview the whole process. This means that a care pathway for this patient group should be established [8, 12].

### History

Not every elderly patient is able to answer questions adequately. For further information the family, nursing home staff and paramedics are important. Be aware of legal responsibilities and patient treatment limitations.

### Diagnostics

Key points: physical examination, basic lab, X-ray.

Basic steps in this process are:

1. General physical examination (documentation: decubitus, mental state, dehydration)
2. Registration of fracture signs
3. Basic lab: haemoglobin, electrolytes, renal function and coagulation
4. ECG
5. Thoracic X-ray (starting point)
6. AP pelvic X-ray and a lateral view if possible [13].

CT-scan is indicated if the X-rays show no fracture but there is a high index of suspicion at physical examination [14, 15]. An MRI is optional for pathological fractures [16].

The differential diagnosis should include:

1. Hip contusion
2. Pelvic fracture (pubic branch)
3. Fracture of the acetabulum
4. Fracture of the femoral head
5. Fracture of the greater trochanter

### Preoperative workup

Key points: management of pain, decubitus, delirium.

At least a trauma (orthopaedic) surgeon, anaesthetist and geriatrician should be involved. Depending on the local situation, one of these medical specialists has to coordinate the workup. Clear interdisciplinary agreements are necessary [8].

Pain management is very important [17, 18]. Apart from analgesic drugs there are several options:

1. Regional block, for example fascia iliaca compartment block [19]
2. Traction splint
3. Urinal catheter

Decubitus prevention has to start at admission. A pressure-relieving mattress is necessary in bed-bound patients [20]. The state of the skin has to be monitored on a daily basis.

Early prophylaxis, diagnosis and treatment of delirium are important [21, 22]. The mental status has to be monitored on a daily basis. One option is using the delirium observational scale (DOS).

To avoid electrolyte disorders and dehydration, fluid management has to start early [23].

Many elderly persons take anticoagulant drugs. Be aware of coagulation disorders. This has to be addressed preoperatively.

### Operation

Key points: conservative treatment, endoprosthesis, intramedullary device, DHS.

#### Conservative treatment

There might be an option for nonoperative treatment in case of a valgus-impacted femoral neck fracture in a vital patient without severe osteoporosis [10]. Depending on pain, weight-bearing has to start early. Before discharge an X-ray control of the hip is advisable [24, 25].

In pertrochanteric fractures there are hardly any indications for conservative treatment. Exceptions are patients with a severe general condition, like ASA 5 patients.

#### Operative treatment

The operation should be performed during the daytime by a dedicated team [26]. Especially for head-preserving procedures it should take place within 24 h. Most important is the general condition of the patient. In patients with a severe general condition the preoperative workup may take more than 24 h. Fasting time should be as short as possible [27].

The choice between regional and general anaesthesia shows no influence on the incidence of perioperative blood loss, postoperative respiratory insufficiency, myocardial infarction, myocardial insufficiency, renal insufficiency or cerebrovascular deficits [28, 29]. Regional anaesthesia in proximal femoral fractures diminishes the risk of thrombosis [30].

Antibiotic prophylaxis has to start 30 min prior to the operation [31]. The operative treatment of femoral neck and pertrochanteric fractures is discussed controversially. The following recommendation is based on the article ‘A new algorithm for hip fracture surgery’ by Palm et al. from Copenhagen and on the German guidelines for femoral neck and pertrochanteric fractures [32, 33].

### Femoral neck fractures



A femoral neck fracture in a head-preserving procedure must be reduced anatomically.

#### Nondisplaced fractures (Garden 1 and 2, <20° posterior tilt)

- Osteosynthesis: dynamic hip screw, cannulated screws or Hansson pins.

#### Displaced fractures (Garden 3 and 4, >20° posterior tilt)

- Prosthesis; the choice between a total hip prosthesis and a hemiarthroplasty depends on the age and general condition of the patient.

Patients from nursing homes with nosocomial infections colonised with MRSA of ESBL have a higher risk of postoperative infection. In these patients with a displaced femoral neck fracture an osteosynthesis can be considered [34–36].

#### Pertrochanteric fracture



Stable fractures (AO/OTA type A.1 and A.2.1).

- Dynamic hip screw.

Unstable fractures (AO/OTA type A.2.2, A.2.3 and A.3).

- Antegrade intramedullary nail.

The aim of all procedures is early full weight-bearing.

### Postoperative treatment

Key points: avoid pulmonary embolism, hypoxemia, delirium, decubitus.

All hip fracture patients should be clustered in one nursing ward in order to increase the experience of the nursing staff, thus improving the quality of care for the patients, and paying special attention to the care of elderly patients (early start of rehabilitation, adequate diet, pressure ulcer prevention).

Multidisciplinary teamwork is generally considered effective in hip fracture rehabilitation. At least a trauma (orthopaedic) surgeon, geriatrician, dietician and physiotherapist should be involved. During the stay at the nursing ward the patients should be visited routinely by the geriatrician [8].

The incidence of thrombosis and pulmonary embolism after a hip fracture is high. The incidence of symptomatic venous thromboembolism is low (1.34 %) in patients given pharmacological thromboprophylaxis [13, 37, 38]. Sequential compression and arterial venous foot impulse systems can reduce the risk of DVT [13, 39]. Mechanical prophylaxis is labour-intensive and poorly tolerated. There is no good evidence that compression stockings reduce the incidence of venous thromboembolism [13, 39]. Pharmacological prophylaxis is recommended [40]. In the majority of cases low molecular-weight heparin (LMWH) is given for 4–6 weeks. Other choices, like coumarin or fondaparinux, are an option.

Adequate pain relief is associated with reduced cardiovascular, respiratory and gastrointestinal problems and a lower incidence of delirium. Several drugs can be used for pain relief. The choice depends on the circumstances in the specific country and hospital. Pain intensity should be scored with the aid of a visual analog scale (VAS score) on a regular basis.

Hypoxemia is a serious postoperative problem and can persist several days after the operation. Routine use of pulse oxymetry can reduce the incidence of hypoxemia. Supplemental oxygen should be given in the first postoperative hours and as long as hypoxemia persists [13, 41].

Fluid and electrolyte management should be monitored routinely [23]. Malnutrition occurs frequently in the elderly. Poor nutrition can lead to mental apathy, muscle wasting and weakness, impairs cardiac function, and lowers immunity to infection. The nursing staff has to assess the nutritional status with the aid of a malnutrition score such as the MUST score. A dietician should be involved. Oral protein feeds provide protein, energy, some vitamins and minerals, and may have a positive impact on postoperative morbidity [42].

Delirium is a serious and frequent postoperative complication and a negative prognostic factor for the outcome. Prophylaxis should start early. Mental state should be monitored frequently with the aid of a delirium attention protocol. Pharmacological treatment has to be started when delirium occurs.

Decubitus prophylaxis has to start early. The patient should be transferred on a pressure-relieving mattress at admission [20]. The nursing staff should estimate pressure sore risk with the aid of a decubitus score.

A urinary catheter should be removed as early as possible.

Early mobilisation can prevent decubitus, thromboembolism and pneumonia. The patient should be mobilised within 24 h [43]. If possible, full weight-bearing should be achieved. Balance and gait are essential components of mobility. Activities of daily living such as transferring, washing, dressing and toileting should be trained. A physiotherapist should be involved in the treatment.

### Discharge, nursing home and outpatient clinic

Key points: early planning, co-working, prevention.

To prevent delay, discharge procedures have to start early. Cooperation with nursing homes and geriatric rehabilitation units should be considered. A nursing home physician should be involved early. The rehabilitation programs of hospitals and rehabilitation units should be coordinated [8].

After a hip fracture the risk of another fracture increases considerably. The multidisciplinary team has to advice and initiate osteoporotic diagnosis and treatment [9, 11, 44, 45]. This depends on the local situation and on who is in charge. Bone density measurement is recommended. In patients above 80 years of age with prevalent hip fracture, the WHO does not advice densitometry to initiate osteoporotic treatment.

Results of fall prevention programs are controversial. Hip protectors are not recommended, as the incidence rate of hip fractures in protected vs. unprotected hips among nursing home residents did not differ (3.1 vs. 2.5 %) [46].

According to Masud’s multifactorial interdisciplinary prevention programmes in the late 90s, post-fall and post-fracture strategies currently focus on multifactorial interventions with osteoporosis and sarcopenia treatment [36, 47]. Lean-mass DXA measurement correlates with body composition DXA scans and depicts the loss of muscle mass [48].
